# Supplementary figures and images for: Cellular co-infection can modulate the efficiency of influenza A virus production and shape the interferon response
Source: PLoS Pathog. 2020 Oct 16;16(10):e1008974. doi: 10.1371/journal.ppat.1008974 (PMC7592918; doi:10.1371/journal.ppat.1008974)

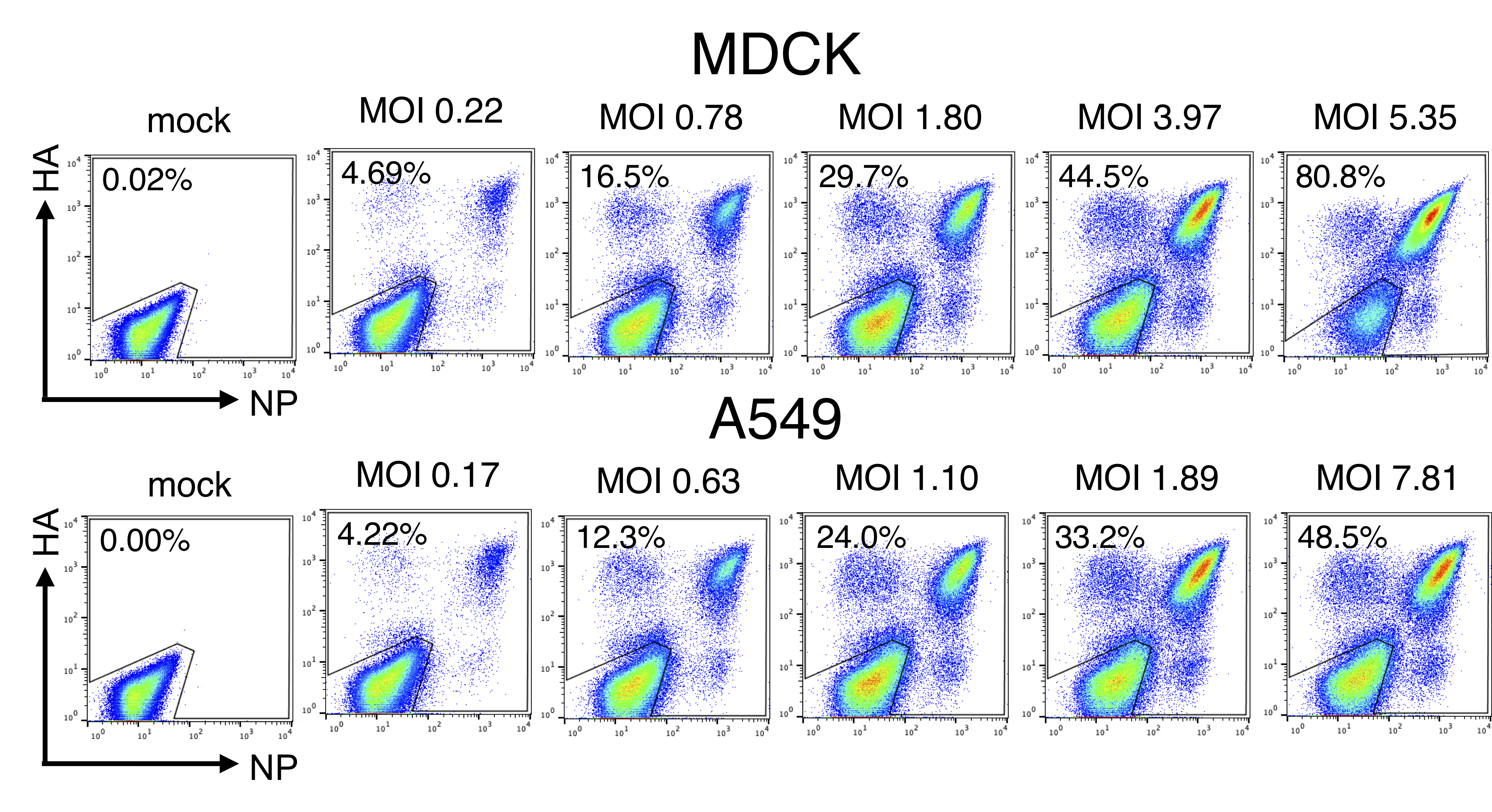

Supplement: S1 Fig — For MDCK (top row) and A549 (bottom row) cells. Gates for determining infection status were drawn based on NP and HA expression of mock cells. Gates were modified by eye for the MDCK cell line at the MOI of 5.35 for MDCK and 7.81 for A549 to better exclude negative cells. These data were generated from the same experiments used to generate cell death and virus production data. (TIFF) [file ppat.1008974.s001.tiff]

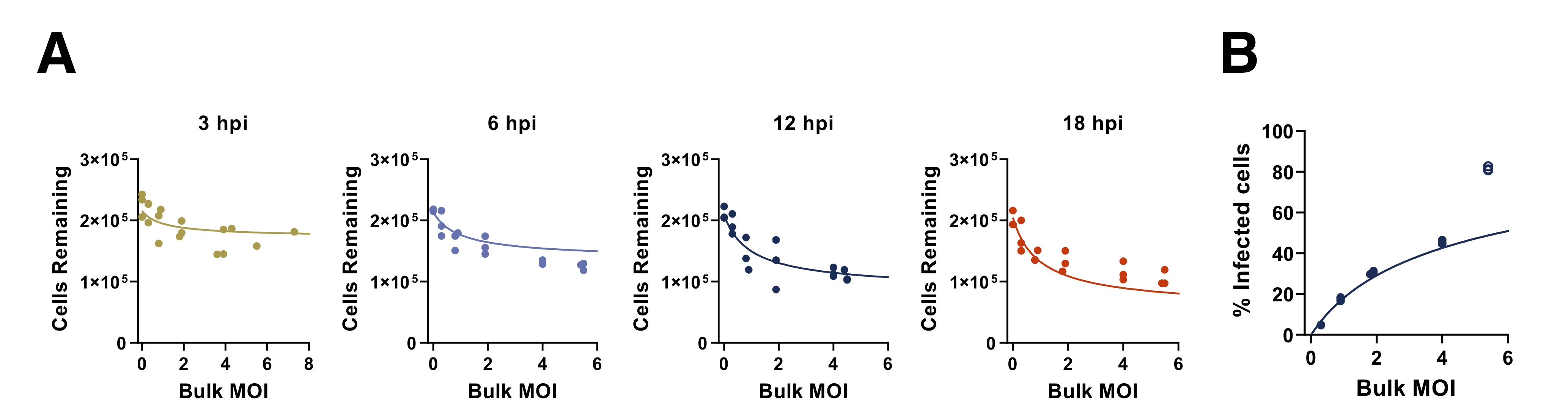

Supplement: S2 Fig — (A) The number of cells remaining for 3, 6, 12, and 18 hpi, respectively, as a function of bulk MOI, along with time-independent, input-independent cell death rate model fits (lines). (B) Number of surviving MDCK cells that are infected at 18 hpi, as measured by FACS, along with the negative binomial distribution model fit (line). As in Fig 2C, statistical parameterization of this model (overdispersion parameter r = 0.756; S1 Table) indicates a high level of overdispersion and significant deviation from a Poisson-distributed model. FACS data at high bulk MOI (open circles) were excluded from model fits due to the lack of confidence in high MOI measurements. (TIFF) [file ppat.1008974.s002.tiff]

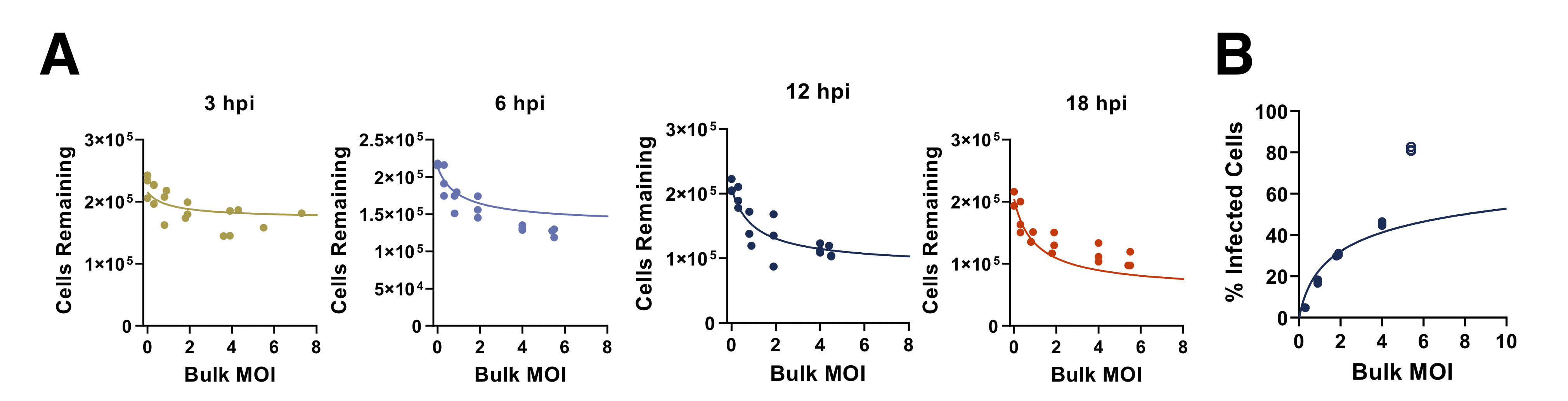

Supplement: S3 Fig — (A) The number of cells remaining for 3, 6, 12, and 18 hpi, respectively, as a function of bulk MOI, along with time-independent, input-dependent cell death rate model fits (lines). (B) Number of surviving MDCK cells that are infected at 18 hpi, as measured by FACS, along with the negative binomial distribution model fit (line). As in Fig 2C, statistical parameterization of this model (overdispersion parameter r = 0.756; S1 Table) indicates a high level of overdispersion and significant deviation from a Poisson-distributed model. FACS data at high bulk MOI (open circles) were excluded from model fits due to the lack of confidence in high MOI measurements. (TIFF) [file ppat.1008974.s003.tiff]

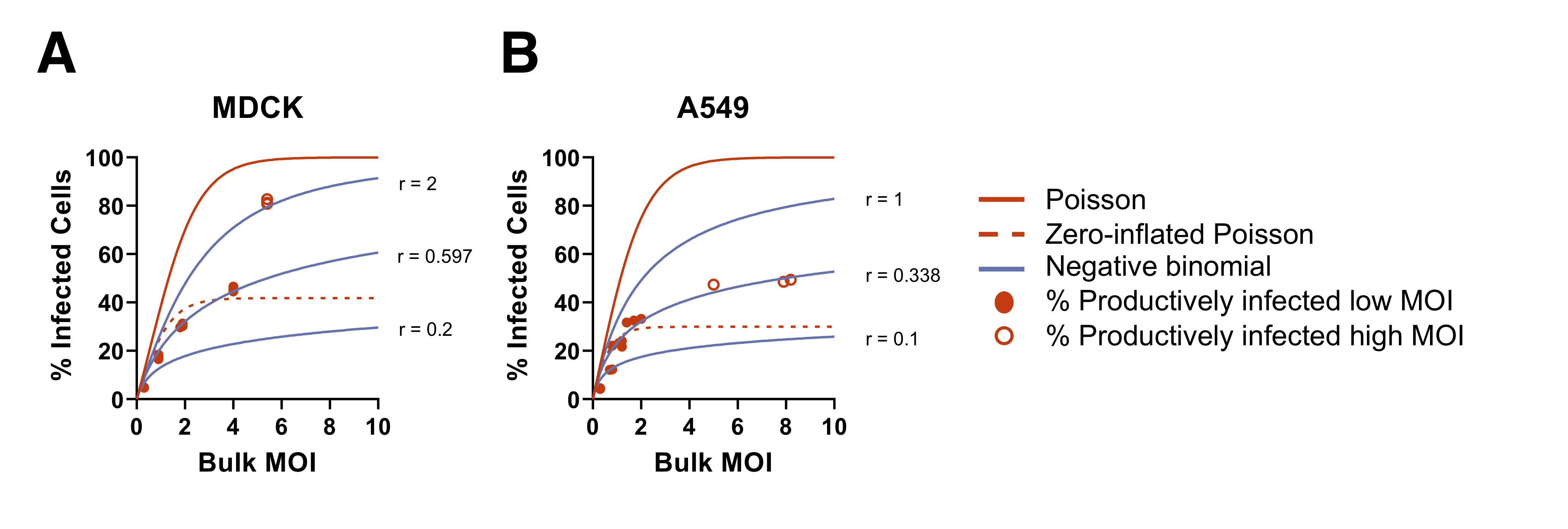

Supplement: S4 Fig — (A) Number of surviving MDCK cells infected at 18 hpi (dots) and viral dispersion model fits to these data (lines). Under the most supported cell death rate model (the time-dependent, input-independent model), the best fit to the FACS data occurred under the negative binomial model with an overdispersion parameter of r = 0.597 (solid orange line; S1 Table). FACS data points from the high MOI experiments (open circles) were excluded from the model fit. Higher levels of overdispersion (r = 0.2; blue line) underestimated percentages of infected cells at 18 hpi. Lower levels of overdispersion (r = 2; blue line) overestimated percentages of infected cells at 18 hpi. To obtain the negative binomial models at fixed dispersion parameter values, r = 0.2, 2, we re-fit the parameters of the time-dependent, input-independent cell death rate model. A Poisson distribution assumption (r = ∞; solid red line) severely overestimated percentages of infected cells at 18 hpi. The zero-inflated Poisson is shown with the time-dependent, input-independent cell death rate model and with the probability of extra zeros, p = 0.312 (dashed red line). S1 Table shows the four cell death rate models parameterized under the assumption of Poisson, negative binomial, and zero-inflated Poisson distributions for viral input across cells. ΔAIC values for these models are significantly larger than 0, indicating that the negative binomial distribution model is strongly preferred over both the Poisson and zero-inflated Poisson distribution models. (B) Number of surviving A549 cells infected at 18 hpi (dots) and viral dispersion model fits to these data (lines). Under the most supported cell death rate model (the time-dependent, input-independent model), the best fit to the FACS data occurred under the negative binomial model with an overdispersion parameter of r = 0.338 (solid orange line; S2 Table). FACS data points from the high MOI experiments (open circles) were excluded from the model fit. Higher leve [file ppat.1008974.s004.tiff]

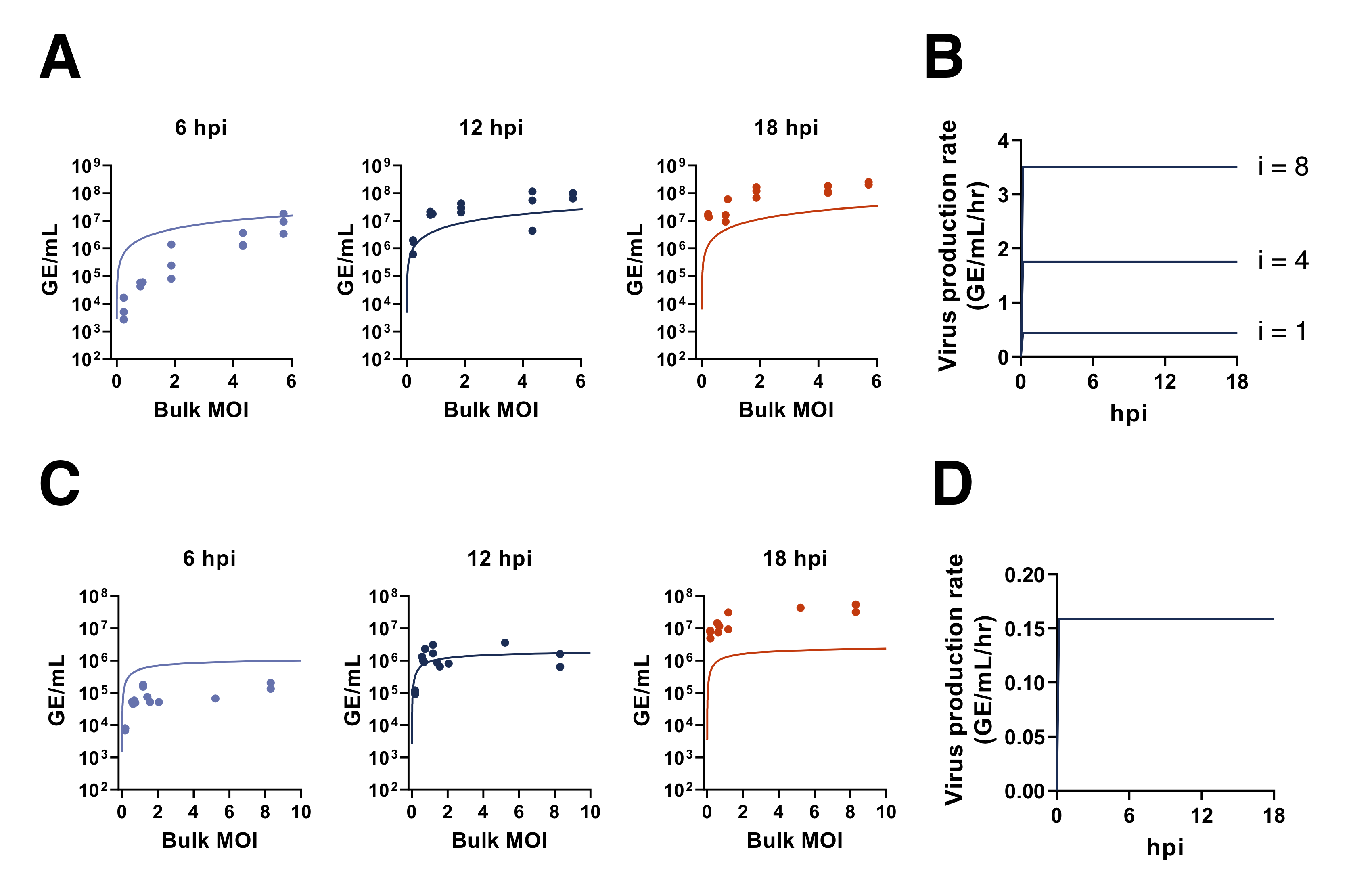

Supplement: S5 Fig — (A) Time-independent, linear input-dependent model fits to virus production in MDCK cells overestimate viral output at 6 hpi and underestimate the output at 18 hpi. (B) The virus production rate is constant over time and the rate increases linearly with increasing cellular MOI: i = 1, 4, 8. (C) Time-independent, input-independent model fits to virus production in A549 cells overestimate viral output at 6 hpi and underestimate viral output at 18 hpi. (D) The virus production rate is constant over time and independent of the cellular MOI. (TIFF) [file ppat.1008974.s005.tiff]

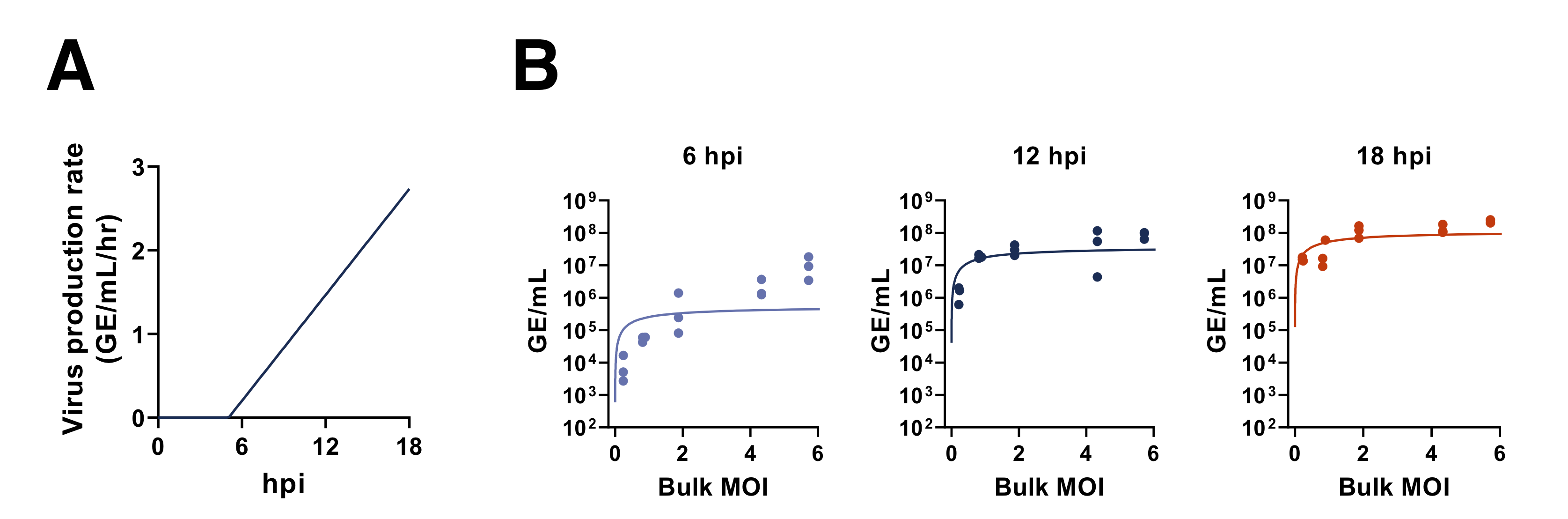

Supplement: S6 Fig — (A) The time delay in virus production was estimated in this model to be 5.27 days. After that point, the virus production rate was assumed to increase linearly in time, with an estimated slope of 2.52. (B) Model fits to virus production in MDCK cells (S3 Table). (TIFF) [file ppat.1008974.s006.tiff]

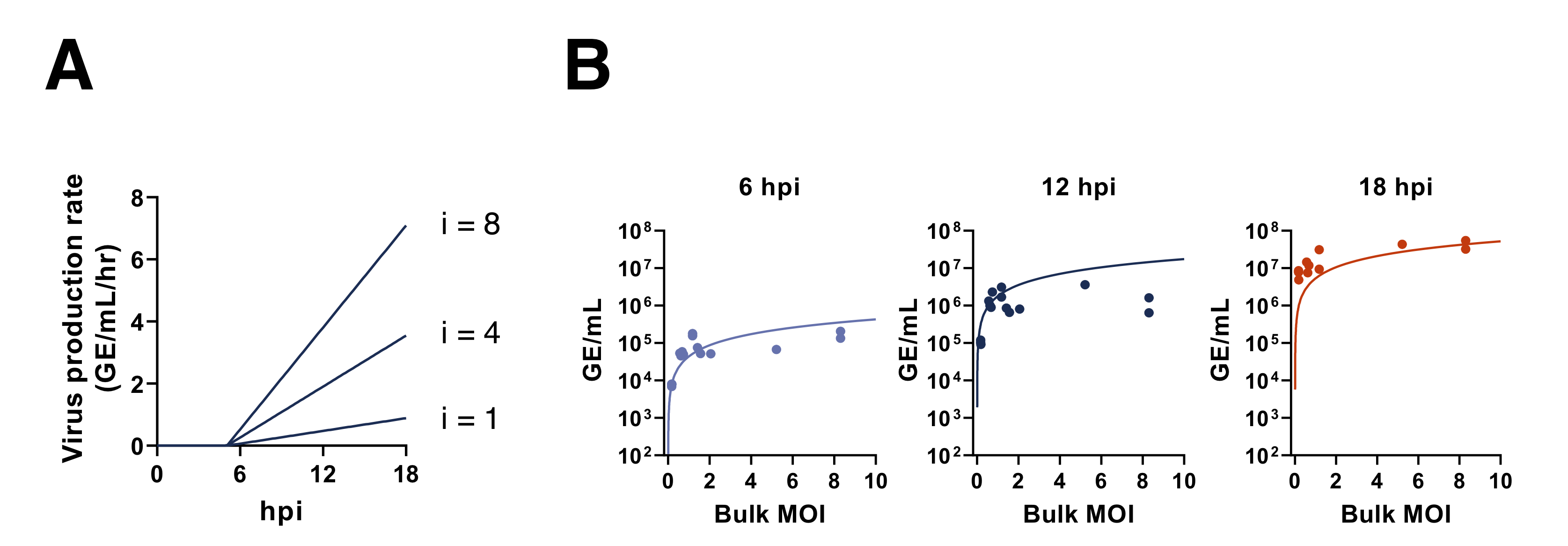

Supplement: S7 Fig — (A) The virus production rate is zero until ~ 5 hpi after which point the rate increases linearly in time. The slope of this linear increase depends on the cellular MOI: i = 1, 4, 8 (S4 Table). (B) The linear input-dependent model fits to data: at 6 and 12 hpi, the model overestimates viral output for high bulk MOI values, and at 18 hpi, the model underestimates viral output for low bulk MOI values. (TIFF) [file ppat.1008974.s007.tiff]

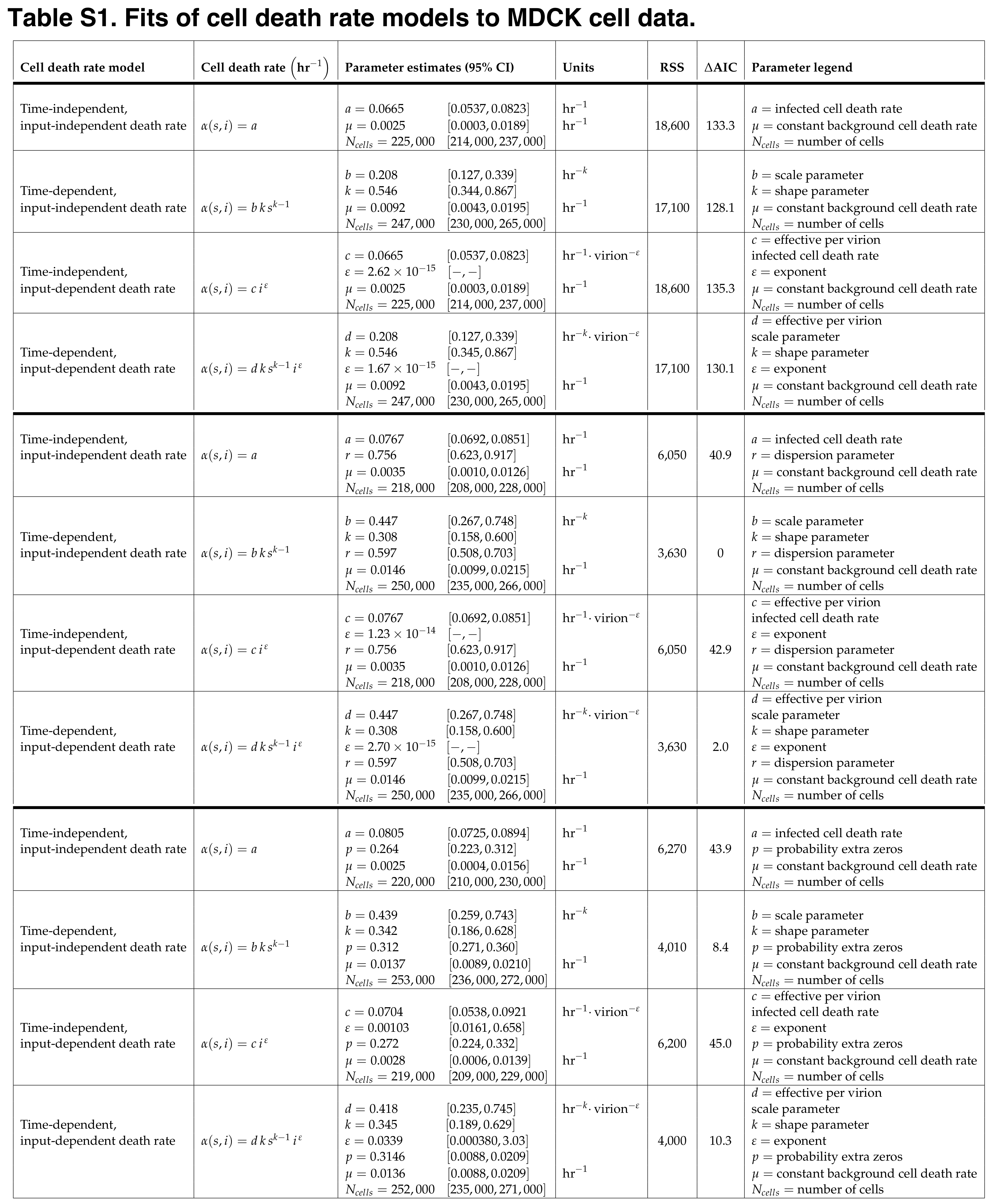

Supplement: S1 Table — Rows correspond to distinct cell death rate models. The mathematical formulation for each cell death rate model is provided in the second column. The models are group by the virus distribution assumption, going from top to bottom: Poisson, Negative binomial, zero-inflated Poisson. Point estimates and 95% confidence intervals are provided in the third column for each model’s parameters. Confidence intervals for parameter estimates close to zero were omitted (Methods). Units of the parameters are provided in the fourth column. The fifth column lists the residual sum of squares (RSS) for each model, parameterized with the point estimates of the third column. The model most supported by the data is the time-dependent, input-independent model (ΔAIC = 0). Models with higher ΔAIC have less statistical support. (TIFF) [file ppat.1008974.s008.tiff]

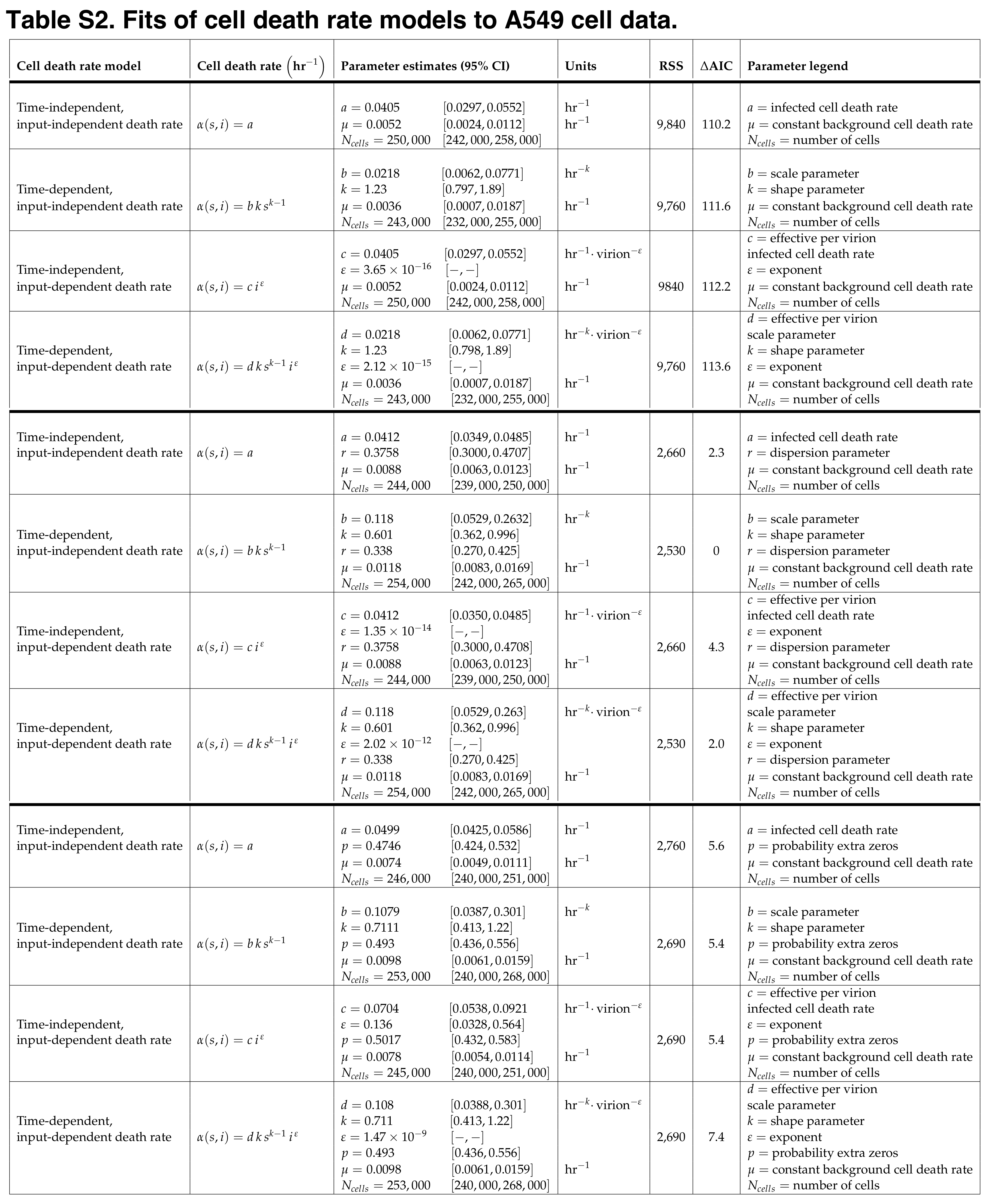

Supplement: S2 Table — As in S1 Table, rows correspond to distinct cell death rate models assuming the viral infection distribution from top to bottom: Poisson, negative binomial, zero-inflated Poisson. The model most supported by the data is the time-dependent, input-independent model (ΔAIC = 0). (TIFF) [file ppat.1008974.s009.tiff]

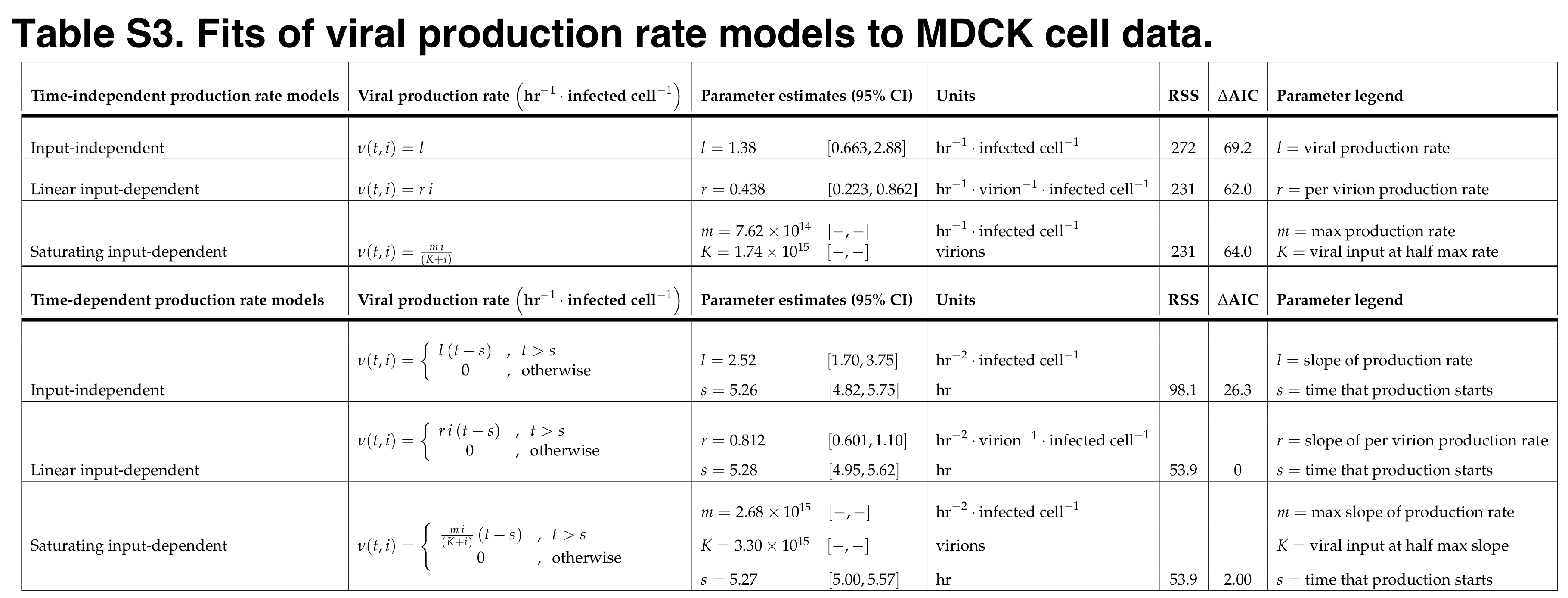

Supplement: S3 Table — Rows correspond to distinct viral production rate models. Parameter estimates are given along with 95 percent confidence intervals for viral production rate model parameters. The model that is most supported by the data has ΔAIC = 0, and models with higher ΔAIC have less statistical support. Confidence intervals for high parameter estimates were omitted (see methods). (TIFF) [file ppat.1008974.s010.tiff]

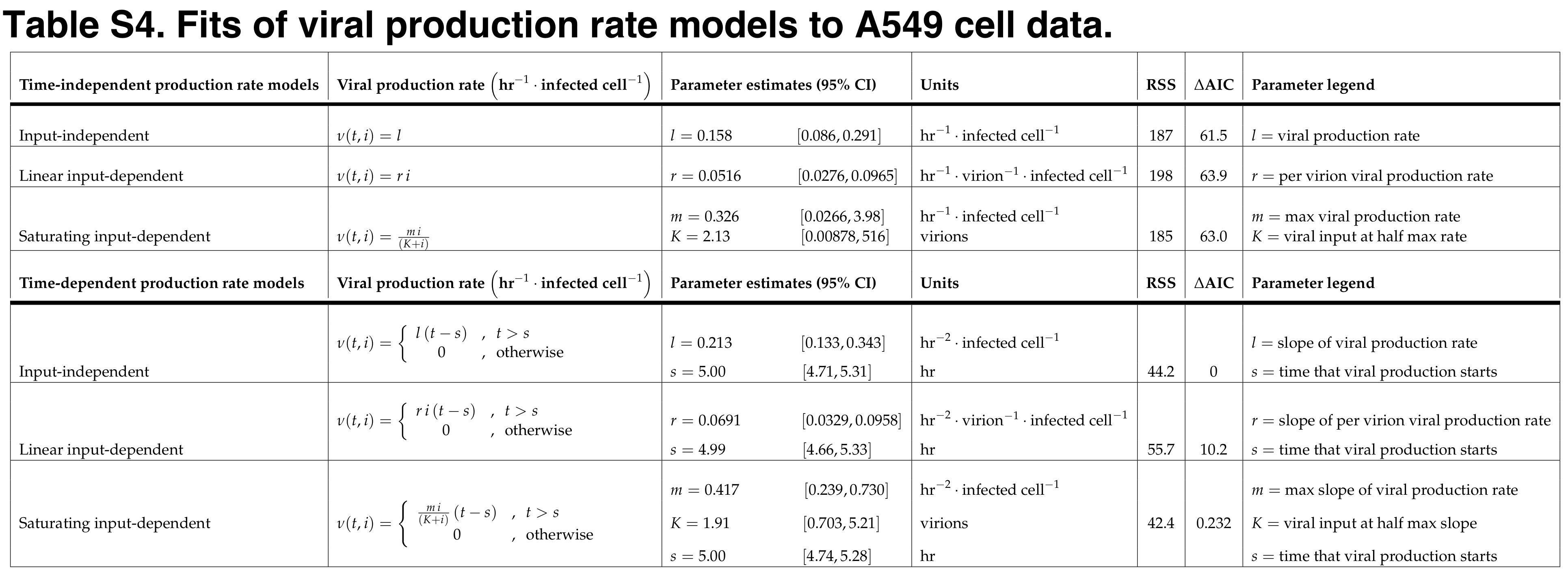

Supplement: S4 Table — Rows correspond to distinct viral production rate models. Parameter estimates are given along with 95 percent confidence intervals for viral production rate model parameters. The model that is most supported by the data has ΔAIC = 0, and models with higher ΔAIC have less statistical support. (TIFF) [file ppat.1008974.s011.tiff]

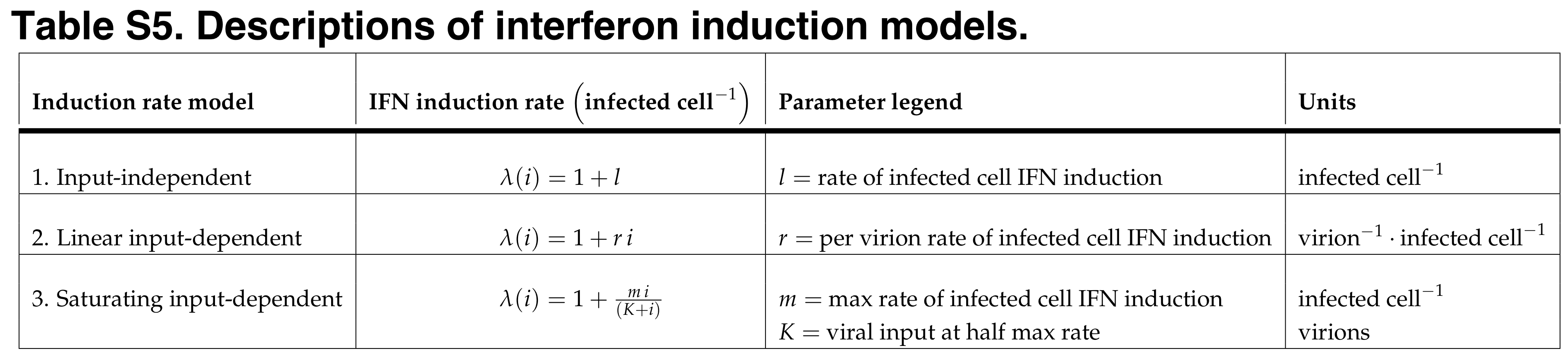

Supplement: S5 Table — We considered three IFN induction models in which the induction rates are independent of time but differ based on the viral input. We fit an input-independent, linear input-dependent, and saturating input-dependent induction rate models to data at 8 and 18 hpi, giving a total of nine model combinations (see S6 Table). (TIFF) [file ppat.1008974.s012.tiff]

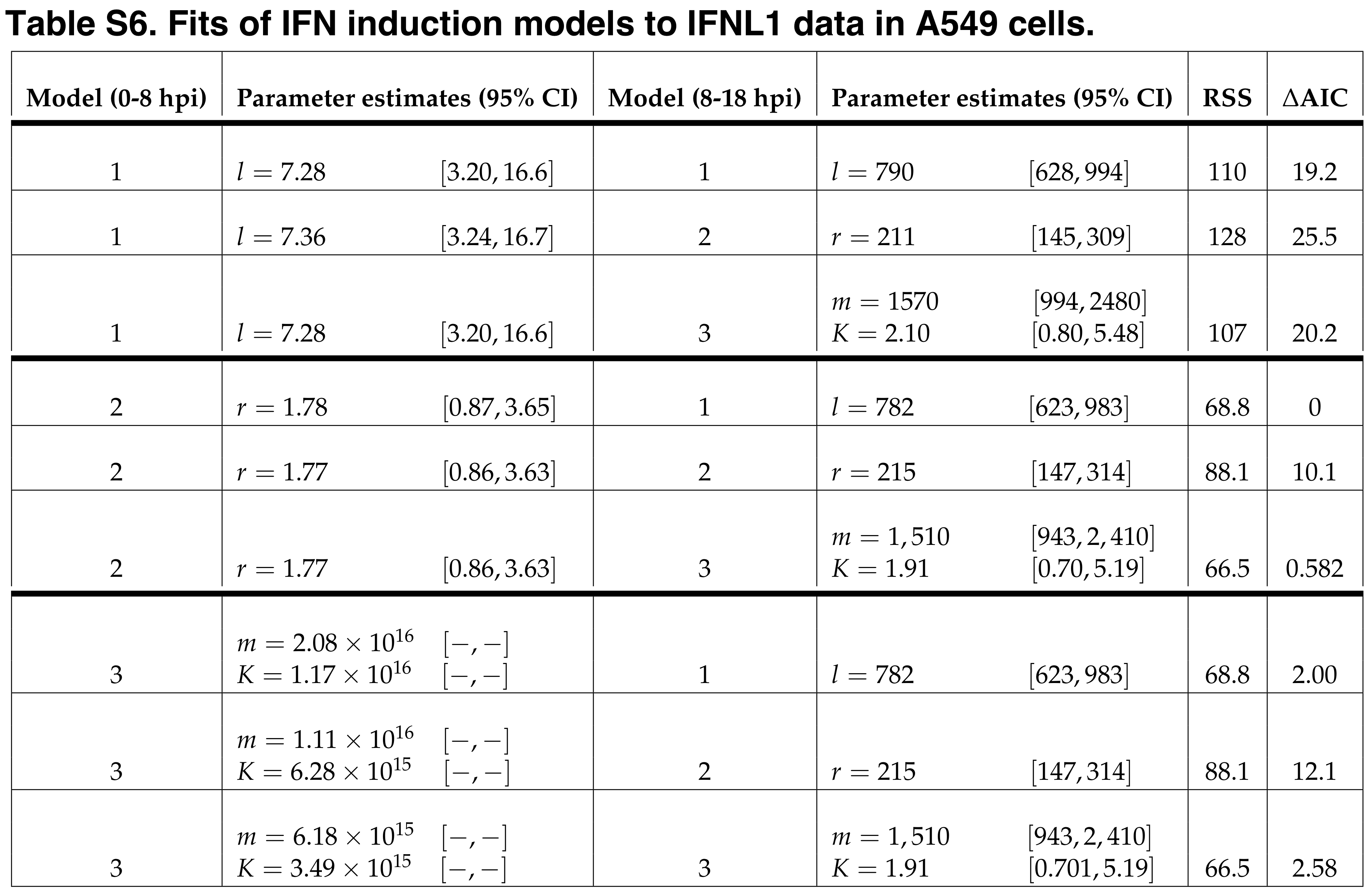

Supplement: S6 Table — Rows correspond to different combinations of the IFN induction rate models listed in S5 Table from 0–8 hpi and from 8–18 hpi. The first column gives the model number from 0–8 hpi, and the third column gives the model number from 8–18 hpi. Parameter estimates are given along with 95 percent confidence intervals for IFN induction rate model parameters. The model that is most supported by the data has ΔAIC = 0, and models with higher ΔAIC have less statistical support. Confidence intervals for high parameter estimates were omitted (see methods). (TIFF) [file ppat.1008974.s013.tiff]

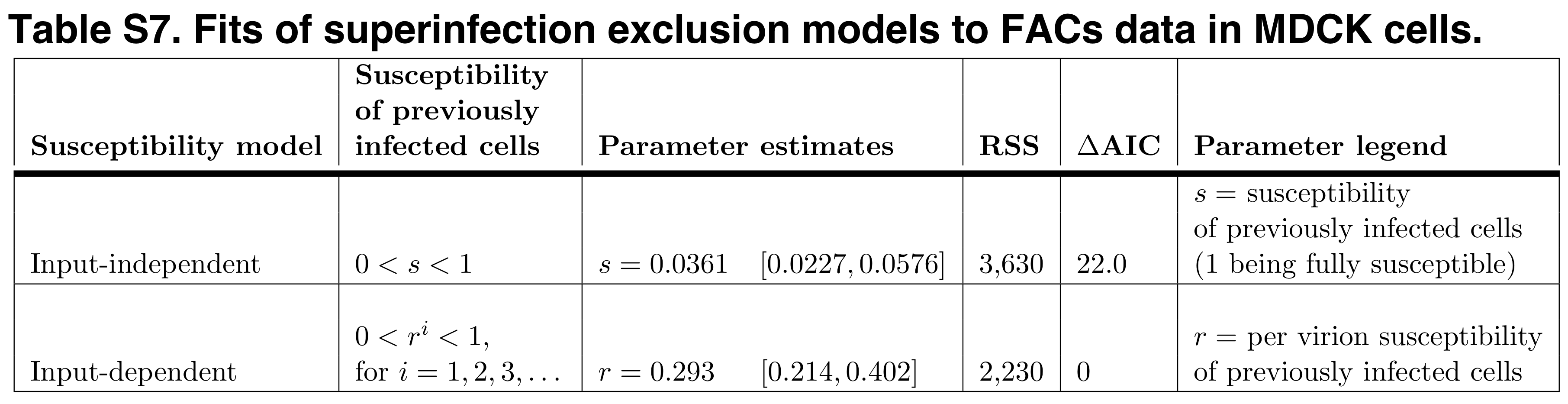

Supplement: S7 Table — Rows correspond to distinct viral production rate models. The first model assumed that all rH3N1-infected cells had the same reduced probability of becoming infected with rH1N2 (input-independent). The second model assumed that the probability of being infected with rH1N2 decreased with cellular rH3N1 MOI (input-dependent). Parameter estimates are given along with 95 percent confidence intervals for viral production rate model parameters. The model that is most supported by the data has ΔAIC = 0, and models with higher ΔAIC have less statistical support. (TIFF) [file ppat.1008974.s014.tiff]

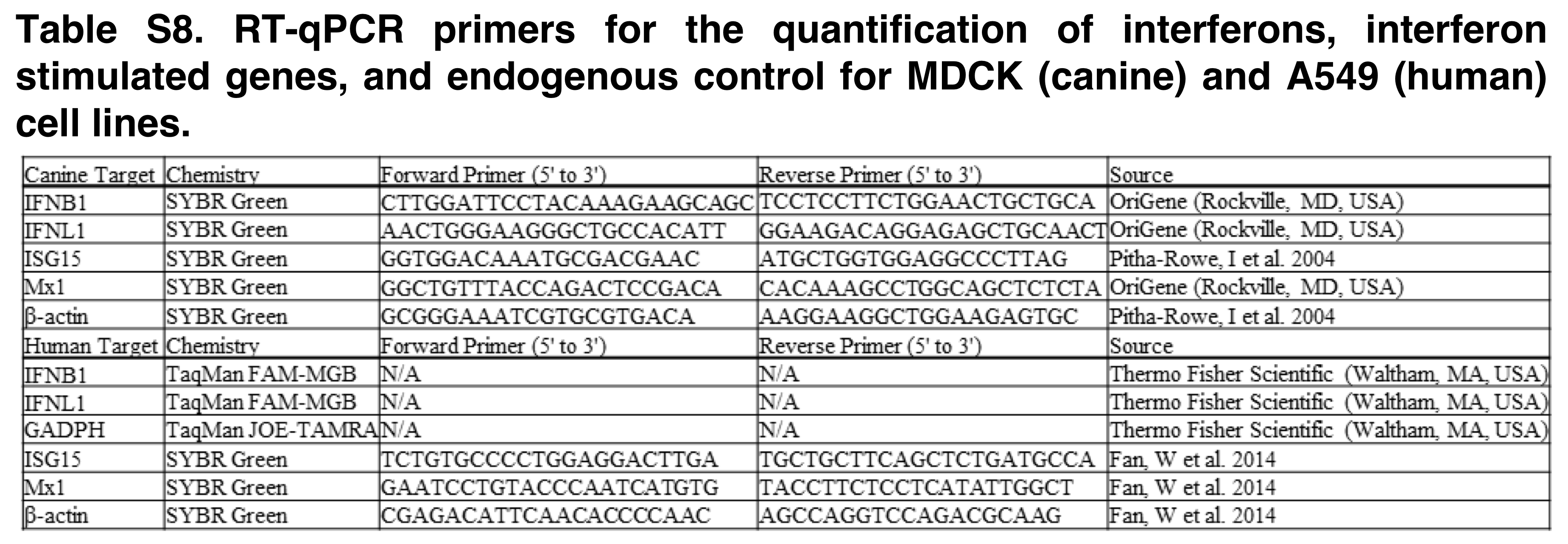

Supplement: S8 Table — SYBR green primers were used for the quantification of canine targets of IFNB1, IFNL1, ISG15, and Mx1, with ß-actin as the endogenous control. For A549, Taqman assays were used for the quantification of IFNB1 and IFNL1 with GADPH as the endogenous control and SYBR green chemistry was used for the quantification of ISG15 and Mx1, with ß-actin as the endogenous control. (TIFF) [file ppat.1008974.s015.tiff]
